# Leveraging imitation learning in agricultural robotics: a comprehensive survey and comparative analysis

**DOI:** 10.3389/frobt.2024.1441312

**Published:** 2024-10-17

**Authors:** Siavash Mahmoudi, Amirreza Davar, Pouya Sohrabipour, Ramesh Bahadur Bist, Yang Tao, Dongyi Wang

**Affiliations:** ^1^ Department of Biological and Agricultural Engineering, University of Arkansas, Fayetteville, AR, United States; ^2^ Department of Mechanical Engineering, University of Arkansas, Fayetteville, AR, United States; ^3^ Department of Bioengineering, University of Maryland, College Park, MD, United States; ^4^ Department of Food Science, University of Arkansas, Fayetteville, AR, United States; ^5^ Center of Scalable and Intelligent Automation in Poultry Processing, Fayetteville, AR, United States

**Keywords:** imitation learning, robotics, agricultural robotics, artificial intelligence, agricultural engineering

## Abstract

Imitation learning (IL), a burgeoning frontier in machine learning, holds immense promise across diverse domains. In recent years, its integration into robotics has sparked significant interest, offering substantial advancements in autonomous control processes. This paper presents an exhaustive insight focusing on the implementation of imitation learning techniques in agricultural robotics. The survey rigorously examines varied research endeavors utilizing imitation learning to address pivotal agricultural challenges. Methodologically, this survey comprehensively investigates multifaceted aspects of imitation learning applications in agricultural robotics. The survey encompasses the identification of agricultural tasks that can potentially be addressed through imitation learning, detailed analysis of specific models and frameworks, and a thorough assessment of performance metrics employed in the surveyed studies. Additionally, it includes a comparative analysis between imitation learning techniques and conventional control methodologies in the realm of robotics. The findings derived from this survey unveil profound insights into the applications of imitation learning in agricultural robotics. These methods are highlighted for their potential to significantly improve task execution in dynamic and high-dimensional action spaces prevalent in agricultural settings, such as precision farming. Despite promising advancements, the survey discusses considerable challenges in data quality, environmental variability, and computational constraints that IL must overcome. The survey also addresses the ethical and social implications of implementing such technologies, emphasizing the need for robust policy frameworks to manage the societal impacts of automation. These findings hold substantial implications, showcasing the potential of imitation learning to revolutionize processes in agricultural robotics. This research significantly contributes to envisioning innovative applications and tools within the agricultural robotics domain, promising heightened productivity and efficiency in robotic agricultural systems. It underscores the potential for remarkable enhancements in various agricultural processes, signaling a transformative trajectory for the sector, particularly in the realm of robotics and autonomous systems.

## 1 Introduction

In an era marked by demographic shifts and escalating food security concerns, the agricultural sector requires transformative solutions to meet increasing demands. The integration of advanced technologies, notably agricultural robotics, has received considerable attention and investment, evident from the growing scholarly focus on these innovations (Cheng et al., 2023). The constraints of traditional farming, marked by labor-intensive processes, imprecise environmental information measurement, and ineffective crop monitoring, underscore the pressing need for more streamlined and sophisticated agricultural practices leveraging artificial intelligence (AI) (Wang, 2019) and robotics (Yépez-Ponce et al., 2023). Researchers are actively working towards creating intelligent, cost-effective, and highly productive agricultural systems that integrate sensor technology and Internet of Things (IoT) (Brewster et al., 2017), data management (Wolfert et al., 2017), decision-making algorithms (Robert et al., 2016), robotics (Fountas et al., 2020), and advanced mechanisms to revolutionize traditional agricultural methods (Wakchaure et al., 2023). This push for innovation is not merely technological but deeply rooted in the urgent global need to enhance agricultural sustainability and food security, reflecting the critical role of agriculture in climate change and its socioeconomic impacts (Grieve et al., 2019). As such, the integration of robotic manipulations into agricultural practices represents a transformative leap, poised to optimize farming methodologies, augment productivity, and address the evolving needs of sustainable food production in the face of growing global demands (Grieve et al., 2019).

In recent years, from the foundational robotic research perspective, the integration of AI in robotic manipulation has undergone a paradigm shift, moving away from traditional predetermined control algorithms towards adaptive, learning-based control methodologies. This evolution, as noted by Yang et al. (2016) and Hussein et al. (2017), reflects the limitations of conventional control algorithm methods, particularly in enabling robots to dynamically adapt to varying tasks and environments. As we delve into the realm of agriculture, the challenges of integration of robotic technology presented are particularly noteworthy, primarily stemming from the inherent variability characterizing agricultural products and unconstrained field conditions. In contrast to industries marked by product standardization, the agricultural domain exhibits substantial diversity in terms of product morphology, including but not limited to variant size, visual features, and tactile textures (Eizicovits and Berman, 2014). The diversity necessitates the development of robotic systems capable of adapting to the intricate variations prevalent in agricultural environments, particularly in tasks such as planting, harvesting, and weeding (Gonzalez-de Santos et al., 2020). These challenges have provided excellent scenarios for the application of learning-based robotic control methodologies.

The general agricultural robotic systems, like other robotic systems, are composed of sensing systems, actuating systems, and control algorithms. For environmental sensing, visual recognition, plays an important role in recognizing and localization of objects of interest while presenting a formidable challenge, necessitating advanced systems capable of discerning subtle differences between the targeted objects and other environmental elements. The considerable variability in color, shape, and size among agricultural products demands the integration of sophisticated visual recognition technologies (Wu et al., 2021). Current research in this domain mainly leverages various Deep Learning (DL) models to enhance the accuracy of visual perception in robotic systems. Convolutional Neural Networks (CNNs), for instance, have been instrumental in processing and analyzing complex visual data, enabling robots to effectively differentiate between various plant species and detect anomalies such as disease or pest infestation (Prakash and Prakasam, 2023; Rezk et al., 2022). Techniques such as transfer learning have also been applied, allowing models trained on extensive datasets to be adapted for specific agricultural tasks by fine-tuning based on relatively small task-specific datasets, thereby improving efficiency and reducing the need for extensive field-specific data collection (Goel et al., 2022; Moiz et al., 2022). In addition to visual sensing, the tactile sensing capabilities equipped by robotic systems enable the robots to potentially handle agricultural products ranging from texture to firmness which pose significant hurdles in the practice, such as for the robotic harvesting tasks (Mandil et al., 2023). Recent advancements have seen the development of various types of tactile sensors, including capacitive, resistive, and piezoelectric sensors, each offering unique benefits in terms of sensitivity and adaptability to different materials and surfaces (Bayer, 2022; Baldini et al., 2023). For example, capacitive sensors, which measure changes in capacitance when in contact with an object, have been effectively used in robotic grippers to ascertain the firmness and ripeness of fruits, enabling delicate handling and minimizing damage (Alshawabkeh et al., 2023).

In practice, addressing the comprehensive challenges in agriculture, such as unpredictable environmental conditions and the need for precise and gentle handling of process like chicken rehanging tasks in the poultry industry, requires a holistic approach in robotic system design. Robotic pollination is also an important consideration for similar environments, where the need for adaptability and precision in delicate tasks, like pollinating crops in large greenhouses, mirrors the intricacies seen in other agricultural operations. (Broussard et al., 2023). As bee populations decline, robots like the PollyPollinator are being developed to autonomously pollinate crops, particularly in large greenhouses (Ohi et al., 2018). Automated greenhouse management is also advancing through the use of imitation learning techniques. Robots like Sweeper are used to autonomously harvest bell peppers, demonstrating the ability to perform delicate tasks in controlled environments (Arad et al., 2020). These systems ensure that optimal growing conditions are maintained while minimizing energy consumption, showcasing the potential of robotics to improve efficiency and sustainability in agriculture. Mentioned tasks, characterized by their complexity and the need for fine motor skills and judgment, are difficult to articulate concisely and standardize across varying conditions. Integrating of multi-sensory data, combining visual and tactile feedback with machine learning algorithms, is a growing area of focus. This multi-sensory integration allows for more nuanced decision-making processes in robots and enhanced dexterity and cognitive abilities, making them capable of performing tasks that traditionally rely heavily on human expertise and adaptability.

Beyond arm manipulation, robotic control critically encompasses the dynamic movement of robotic vehicles. The agricultural landscape, with its uneven and unpredictable terrain, poses significant navigation challenges that traditional wheeled or tracked robots might not efficiently overcome. These challenges necessitate the development of robots with enhanced mobility capabilities to navigate diverse topographies, including slopes, rough surfaces, and areas with soft soil that could impede traditional means of locomotion (Botta et al., 2022). In response to these challenges, legged robotic control emerges as a promising solution, offering superior adaptability and mobility in complex agricultural environments. Legged robots, inspired by the locomotion of animals, can traverse obstacles, step over gaps, and adjust their body configuration to maintain stability on uneven terrain (Yang et al., 2023). This adaptability is crucial in agriculture, where the robots must operate in fields with variable soil types, undulating surfaces, and around crops planted in irregular patterns. Recent advancements in legged robotics, fueled by sophisticated control algorithms and sensory feedback systems, have significantly improved their efficiency and robustness. Techniques such as reinforcement learning and bio-inspired control strategies have been applied to optimize legged locomotion, enabling these robots to make real-time decisions based on environmental feedback. This allows for precise movement control, essential for tasks such as targeted spraying, soil analysis, and crop monitoring, minimizing the risk of damaging the crops or soil structure (Kaur and Bawa, 2022; Sun et al., 2023).

Traditional control strategies in robotics, such as PID (Proportional-Integral-Derivative) controllers and model predictive control (MPC), have been instrumental in enabling precise and reliable actions in structured environments. However, as mentioned above, the agricultural domain presents unique challenges that often exceed the capabilities of these traditional approaches (Li et al., 2023). The inherent variability of natural environments, coupled with the need for delicate handling of agrifood products and navigation through unstructured terrains, calls for more advanced and flexible control strategies.

As these challenges are intricately interconnected, The development of robotic solutions for agriculture epitomizes the quintessence of interdisciplinary collaboration, requiring a confluence of expertise from robotics, computer vision, AI, and agricultural sciences. Such a coalition is indispensable for engendering systems that are not merely adaptable and robust but also keenly attuned to the multifarious demands of agriculture. This is where Imitation Learning (IL) emerges as a particularly valuable tool mimicking expert human behaviors, IL enables robots to assimilate complex tasks with remarkable efficiency, significantly curtailing the need for extensive programming (Yamin and Bhat, 2022). The indispensability of IL in agricultural robotics is underscored by its ability to circumvent the limitations of traditional control mechanisms. IL facilitates the transfer of human expertise and human-like dexterity to robotic systems, empowering them to perform intricate tasks such as precision planting, targeted pesticide application, selective harvesting, and, notably, complex food processing operations. This approach is instrumental in enhancing the adaptability of robots to dynamic agricultural and food processing environments, where precision and delicacy are paramount. By optimizing resource utilization and minimizing environmental impact, IL-based robotic systems represent a promising strategy for enhancing productivity and sustainability in the agriculture and food processing sectors (Krithiga et al., 2017). Furthermore, the integration of IL within agricultural robotics exemplifies the synergistic potential of combining machine learning with other technological advancements in AI and computer vision which paves the way for enlarging the application scope of the advanced robotic techniques in precision agriculture (Daaboul et al., 2019).

This paper explores the application of IL within the domain of agricultural robotics, showcasing how the synergistic integration of robotics, AI, and agricultural sciences—enhanced through IL—signifies a revolutionary shift in the field. It emphasizes the critical need for evolving the broader landscape of IL research to address the intricate challenges faced by the agricultural sector, thereby enabling robotic systems to play a pivotal role in the advancement of sustainable agriculture.

Hussein et al. (2017) present a seminal survey that systematically categorizes various IL methods, detailing the diversity of learning strategies deployed across multiple domains. This comprehensive review is particularly invaluable as it traces the evolution of IL, outlining key methodological advancements and conceptual frameworks that have shaped the field. Their discussion extends beyond mere categorization, providing critical insights into how different approaches address specific computational challenges, thereby serving as a foundational reference for understanding the progression and refinement of IL technologies.

In a more recent survey, Zare et al. (2024) offer a forward-looking perspective by examining the latest algorithmic advancements and identifying persisting challenges within IL up to the year 2024. Their survey not only synthesizes recent developments but also emphasizes the application of IL in dynamically complex environments. Their findings are especially relevant to agriculture, where IL must adapt to variable and unpredictable conditions, thus highlighting potential areas for technological interventions to enhance precision farming practices. Torabi et al. (2019) delve into the subfield of IL from observation, which becomes crucial in scenarios where explicit action data is limited or absent. This aspect of IL is highly pertinent to agriculture, where capturing detailed action data can be challenging. The review paper explores state-only learning approaches, discussing their practical applications and theoretical underpinnings. The adaptability of these methods to agricultural settings offers significant potential for developing autonomous systems that learn from observational data alone, thus simplifying the integration of IL in field operations.

In more specialized application of IL, Le Mero et al. (2022) review IL techniques for autonomous vehicles, focusing on end-to-end learning for autonomous navigation systems. Although their primary context is vehicular automation, the methodologies they discuss are adaptable to autonomous agricultural machinery, potentially enhancing efficiency in field operations. Fang et al. (2019) delve into robotic manipulation, an area that involves acquiring fine motor skills from human demonstrations. Their insights are particularly applicable to robotic systems that might be employed in precision agriculture tasks such as planting or harvesting.

While the reviews mentioned above offer an extensive overview of imitation learning across diverse disciplines and technical dimensions, it’s essential to underscore the unique orientation of our work. Moving beyond the technical intricacies, our review delves into how these methodologies can be specifically adapted and applied to agricultural contexts. This tailored approach isn’t just about exploring theory; it involves a deep dive into practical applications that are directly relevant to the challenges and needs of the agricultural sector. By bridging theoretical insights with real-world practices, our review aims to provide a practical toolkit for agricultural practitioners and innovators, helping them to harness these advanced techniques in ways that are both effective and meaningful for their specific fieldwork.

The remainder of the paper is organized as follows: Section 2 outlines the search criteria employed to identify relevant articles for this review, detailing both the inclusion and exclusion parameters. Section 3 provides an overview of IL, explaining the key concepts, algorithms, and their specific adaptations for agricultural applications. In Section 4, case studies and practical implementations have been presented that illustrate the successful application of IL in various agricultural settings. Section 5 addresses the challenges and limitations of current IL approaches, offering a critical analysis of what is needed to overcome these barriers. Finally, Section 6 outlines the future directions for research in IL within agriculture, proposing potential innovations and improvements that could further enhance the efficiency and effectiveness of robotic systems in this vital sector.

## 2 Methodology of literature selection

A thorough literature search was conducted across three prominent databases: Web of Science, Science Direct, and Google Scholar, covering studies published from 1985 to 2024. The search focused on imitation learning and its application in agriculture. Many research papers on imitation learning models are available, but no critical review paper is available based on agriculture; therefore, this review holds significant importance. It was searched using special keywords like imitation learning, imitation learning in agriculture, agricultural robotics, precision agriculture, applied artificial intelligence, and smart agriculture. Boolean operators (“AND” and “OR”) were used to refine and narrow the search results, ensuring the retrieved literature was highly relevant. Each abstract was meticulously reviewed to align with the study’s objectives, which centered on two key areas within imitation learning models: data collection and real-time applications. The search was tailored to include papers that employed imitation learning models in agricultural contexts or provided methodological insights into these models.

**FIGURE 1:**
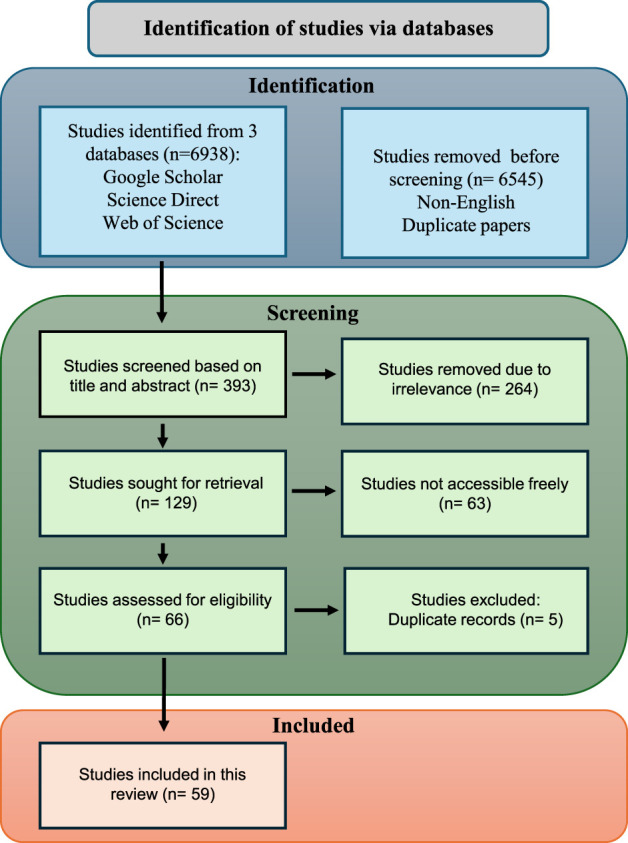
Prisma flow diagram illustrating the literature screening and selection process.

Our review prioritized papers utilizing imitation learning models to achieve real-time learning applications or develop efficient data collection. This paper employed automation tools within the database search engines to streamline and filter the search results, ensuring a rigorous and efficient search process. Only studies published in English were considered, while conference papers and literature from unrelated domains were excluded to maintain focus. In total, 59 research articles were selected for inclusion in this systematic review, providing a comprehensive overview of current advances and applications of imitation learning in agriculture. A review of the existing literature followed the guidelines set by the Preferred Reporting Items for Systematic Reviews and Meta-Analyses (PRISMA) (Page et al., 2021) and is self-explained in Figure 1.

## 3 Foundations of imitation learning

This section provides a comprehensive overview of IL, situating it within the broader context of machine learning and robotics. It emphasizes IL’s distinctive methodology, which allows robotic systems to acquire complex behaviors by observing expert practitioners. Our objective is to explore in depth the fundamental aspects of imitation learning, examining its principal methodologies and principles. We will also discuss the significant impact these techniques have on the advancement of agricultural robotics. A thorough analysis of various imitation learning strategies will reveal how these methods enable robots to learn skills effectively, thereby allowing them to execute intricate agricultural operations with enhanced precision and efficiency.

### 3.1 Introduction to imitation learning

IL introduces a pivotal development in the fields of machine learning and robotics, characterized by its unique method of behavioral acquisition from expert demonstrations. Unlike traditional supervised learning, which depends on discrete labels, or reinforcement learning, which is founded on trial-and-error, IL enables robots to learn complex behaviors by closely observing human expertise (Argall and Billard, 2010). This methodological advancement is essential for applications that require adaptability and precision, particularly in dynamic environments where traditional control methods are inadequate (Schaal, 1999).

**FIGURE 2:**
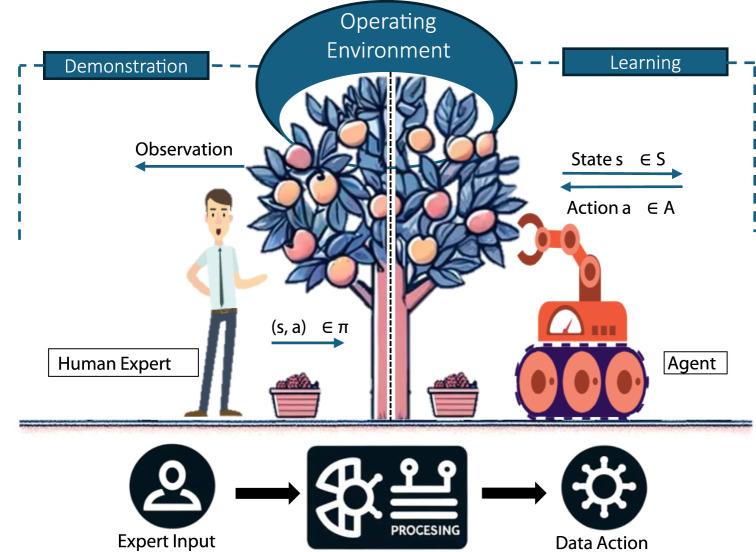
Mapping expertise: A simplified visual representation of imitation learning in action.

The evolution of IL from its conceptual inception to its current applications in advanced robotics showcases the shift from simpler methods to increasingly complex strategies. Initially, IL was primarily about simple behavior mimicry, as demonstrated in Pomerleau’s work (1988), where robots learned actions directly from expert demonstrations without understanding the underlying intentions or rewards. Recent advancements include sophisticated models like Generative Adversarial Imitation Learning (GAIL). In GAIL, a generator (the imitating agent) learns to mimic actions indistinguishably from an expert, under the scrutiny of an adversarially trained discriminator (Ho and Ermon, 2016). Building on the principles of GAIL, newer methods, such as diffusion policy models have emerged. These models integrate various learning techniques to enhance the adaptability and efficiency of IL, enabling robots to perform in more dynamic and unpredictable environments by effectively merging multiple policies into a coherent strategy (Chi et al., 2023). This progression underscores the growing significance and complexity of IL in the field of advanced robotics. Figure 2 shows a simple flow from expert input data to the agent’s learning process, indicating the interactions between state, observation, and action, as guided by an expert’s demonstrated policies.

**FIGURE 3:**
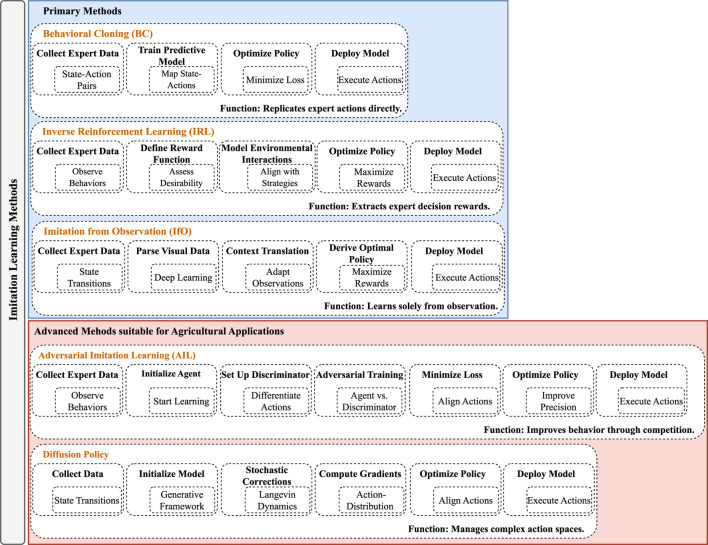
Overview of primary and advanced imitation learning methods for agricultural applications.

IL encompasses a range of strategies essential for advancing robotics, notably Behavioral Cloning (BC), Inverse Reinforcement Learning (IRL), and Imitation from Observation (IfO). In Figure 3, a comprehensive visual comparison of imitation learning methods is presented by categorizing them into two groups: primary methods and advanced methods tailored for agricultural applications. The figure highlights each method’s respective process, starting from data collection to policy deployment, while also providing detailed insights into the unique features of each method.

BC involves the direct replication of behavior by learning from expert actions in specific states, while IRL focuses on deriving underlying reward functions from observed expert behaviors, aiming to understand and replicate the motivations behind these actions. IfO allows for learning without access to expert actions, instead relying solely on observing the state transitions made by the expert. Additionally, advanced methods such as Generative Adversarial Imitation Learning (GAIL) and Diffusion Policy expand the toolkit available for tackling unique challenges in robotics applications, particularly in agriculture. GAIL utilizes adversarial processes to train policies that can not only mimic but also generalize from expert behavior, whereas Diffusion Policy integrates multiple policy models or data sources to create a robust consensus policy, enhancing performance in complex agricultural environments. These advanced techniques help solidify imitation learning as a cornerstone of modern robotics innovation, especially in domains requiring sophisticated interaction with the environment.

### 3.2 Primary methods

#### 3.2.1 Behavioral cloning

The transformative potential of IL in agriculture is its capability to introduce automation and data-driven decision-making into traditional farming practices. BC stands out for its direct and efficient approach, excelling in learning and replicating expert behaviors through a method akin to supervised learning. This method entails constructing a predictive model that maps environmental states to corresponding expert actions, utilizing a dataset of state-action pairs meticulously documented from expert performances. In practice, states can be described by various factors, including soil moisture levels, plant growth stages, or weather conditions, providing a comprehensive context for the robot’s actions. These pairs serve as a model blueprint, guiding it to emulate expert decision-making processes under the assumption that a sufficiently diverse and comprehensive dataset can enable the model to generalize well to new, unseen states (Pomerleau, 1988; Ross et al., 2011). BC’s inherent simplicity, deriving from its problem space abstraction, liberates it from the complexities of environmental dynamics. This characteristic grants BC high adaptability and precision, allowing for its application across a wide range of agricultural tasks by learning from visual and tactile cues and corresponding human expert demonstrations.

The advantage of BC in agriculture lies in its capacity to function independently of complex environmental intricacies. This facilitates its deployment in a variety of crucial agricultural operations. For example, in precision irrigation, BC enables robots to learn from expert demonstrations on when and where to irrigate, considering factors like soil moisture content and weather predictions. By observing the timing, amount, and methods used by experts in different conditions, robots can make data-driven irrigation decisions, optimizing water usage for crop health and yield without constant human supervision. Similarly, in autonomous pest control, BC can teach robots to identify and respond to pest infestations based on visual cues and expert interventions. Through learning from experts’ actions, such as the application of pesticides or the removal of infected plants, robots can autonomously navigate fields, detect pest activity, and apply precise interventions. This not only increases the efficiency and effectiveness of pest control measures but also reduces the reliance on manual labor and the potential for human error.

Both applications exemplify how BC, by distilling complex decision-making processes into learnable patterns from expert demonstrations, contributes significantly to the enhancement of modern farming operations. These implementations of BC in agriculture align with the sector’s growing need for real-time, data-driven decision-making, underscoring the methodology’s adaptability and potential to revolutionize traditional farming practices (Kim et al., 2023; Zhang C. et al., 2023).

The objective central to BC is to learn a policy π that emulates expert behavior by minimizing the discrepancy between predicted and expert actions in identical states, formalized in Equation 1:

$$\underset{\pi }{\min}\frac{1}{N}\overset{N}{\underset{i=1}{\sum }}\ell \left(\pi \left(s_{i}\right),a_{i}\right)$$ (1)

Here, N represents the total number of state-action pairs from expert demonstrations, where each pair consists of a state $s_{i}$ and the corresponding action $a_{i}$ taken by the expert. The action $a_{i}$ can range from simple binary decisions (e.g., turn on or off the irrigation) to complex continuous actions (e.g., adjusting the amount of pesticide to distribute based on pest density). The loss function $\ell \left(\pi \left(s_{i}\right),a_{i}\right)$ quantifies the discrepancy between the policy’s predicted action $\pi \left(s_{i}\right)$ and the expert’s actual action $a_{i}$ for each state. A commonly used loss function in BC is the mean squared error (MSE) for continuous operations which have a series of state-action pairs. MSE calculates the square of the difference between predicted and actual actions. For categorical actions, a cross-entropy loss function might be used, measuring the dissimilarity between the predicted probability distribution over actions and the distribution representing the expert’s action.

By effectively minimizing this loss, BC enables the model to closely approximate expert behavior given a specific environmental state, thereby facilitating a direct learning approach from expert demonstrations (Pomerleau, 1988). This process underscores BC’s practicality and efficiency in transmitting human expertise to robots, enhancing their capability to perform complex agricultural tasks with a level of precision and adaptability that mirrors human performance.

Despite BC’s advantages in automating complex tasks by mimicking human behaviors, it confronts the covariate shift problem, a significant challenge when transitioning from training to deployment in more general practical settings. The BC model, trained under expert-generated states such as those from simulated environments, controlled settings, or limited training data, struggles to adequately perform or recognize out-of-distribution states in real agricultural environments. These discrepancies can lead to safety and precision issues, as the model may not have been exposed to the full range of real-world variations during its training phase (Zhou K. et al., 2022; Reddy et al., 2019).

Addressing the covariate shift in BC requires exploring strategies beyond conventional BC methods. These strategies include Interactive Imitation Learning (IIL), expert policy support estimation, and the development of constrained operational domains (Chang et al., 2021). Each strategy aims to overcome BC’s inherent limitations, promoting the deployment of robust and adaptable models suitable for the dynamic agricultural environment.

IIL, with Dataset Aggregation (DAgger) as a pioneering method, minimizes discrepancies between training and testing environments by integrating expert feedback into the learning process, enabling adaptive learning that gradually incorporates real-world feedback (Ross et al., 2011; Ross and Bagnell, 2014). This method can be described in Equation 2 as:

$$\pi_{DAgger}=\arg\underset{\pi }{\min}\overset{N}{\underset{i=1}{\sum }}E_{\left(s,a\right)∼\pi_{i}}\left[\ell \left(\pi \left(s\right),a\right)\right]$$ (2)

The equation represents the optimization process to find the best policy ( π ), which minimizes the expected loss ( ℓ ) over a series of iterations ( N ). Here, $E_{\left(s,a\right)∼\pi_{i}}\left[\ell \left(\pi \left(s\right),a\right)\right]$ calculates the expected loss of actions ( a ) taken in states ( s ) as predicted by the current iteration of the policy ( π ), compared to the expert actions. This approach underscores the model’s continuous adjustment and learning from the discrepancies between its actions and those of the expert, aiming to reduce these differences over time. However, this method introduces challenges, notably the increased cognitive load on human experts, who must provide continuous feedback to refine the model’s accuracy and effectiveness. The requirement for sustained expert involvement can be demanding and may necessitate strategies to manage this aspect efficiently (Li et al., 2022; Kelly et al., 2019; Zhang and Cho, 2017; Hoque et al., 2021).

The estimation of expert policy’s support focuses on defining reward structures that encourage the emulation of expert behavior, which is crucial for precision agricultural tasks (Reddy et al., 2019). Meanwhile, constrained operational domains can be formalized in Equation 3 to ensure that agricultural machinery operates within parameters well-represented in the training data, minimizing the risk of encountering unpredictable states (Dadashi et al., 2020):

$$\underset{\pi }{\min}L\left(\pi \right)\;\text{subject to}\;s\in S_{safe}$$ (3)

Here, L(π) represents the loss function for the policy π , and $S_{safe}$ denotes the set of states considered safe or well-represented in the training data. By constraining the robot to operate only within these predefined safe states, we significantly reduce the likelihood of encountering unpredictable situations that could lead to errors or inefficiencies, thereby enhancing the overall reliability and safety of agricultural operations (Mitka, 2018). This comprehensive approach, supported by a robust theoretical foundation and empirical validation, enhances BC models to suit the complex and variable nature of agricultural environments, ensuring efficiency, safety, and operational fidelity.

In addressing the covariate shift challenge, particularly pertinent to applications like autonomous tractors and drones in agriculture, the augmentation of the imitation loss with additional constraints has proven effective (Bansal et al., 2018). Incorporating synthetic perturbations into the expert’s trajectory exposes the model to non-expert behaviors, such as potential near-collision scenarios, which is crucial for the model to learn avoidance behaviors, thereby enhancing operational safety in agricultural tasks. The augmented loss function is given by Equation 4 as below:

$$L_{total}=L_{imitation}+\lambda \left(L_{safety}+L_{perturbation}\right)$$ (4)

Here, $L_{imitation}$ denotes the standard imitation loss, focusing on replicating the expert’s actions. $L_{safety}$ penalizes actions leading to unsafe outcomes, and $L_{perturbation}$ introduces controlled deviations from the expert’s trajectory to simulate unexpected scenarios. The weighting factor λ plays a critical role in balancing these contributions, ensuring that the model’s training is comprehensive and geared towards operational safety in agricultural contexts.

Learned error detection systems enhance the safety of autonomous agricultural robots by ensuring operations stay within previously demonstrated safe limits. These systems identify potential failure states, restricting actions to prevent errors in essential tasks like crop monitoring and pest control (Wong et al., 2022; Spykman et al., 2021). For crop monitoring, safety constraints might prevent contact with plants to avoid damage. In pest control, constraints ensure precise pesticide application to protect non-target plants and beneficial insects. Such systems proactively mitigate risks by limiting robot actions based on learned safe behaviors, thus safeguarding both crops and the environment.

To enhance the adaptability of BC models in agricultural robotics, it is crucial to address the challenges of learning in the field. Agriculture involves diverse tasks and environmental conditions, necessitating rapid learning from minimal examples. However, comprehensive human demonstrations for training are often constrained by the availability and variability of data, which will be discussed in detail in Section 4. Techniques like Model-Agnostic Meta-Learning (MAML), offline imitation learning, and implicit BC models are vital for enabling robots to adapt quickly, learn from varied datasets, and perform effectively in complex agricultural environments.

MAML emerges as a solution, significantly enhancing a robot’s adaptability with its efficient approach to learning from a few instances. MAML’s optimization formula (Equation 5) is key to its success:

$$\theta^{\prime }=\theta -\alpha \nabla_{\theta }L_{T}\left(f_{\theta }\right)$$ (5)

In this formula, $\theta^{\prime }$ are the model parameters post-update, θ are the initial parameters, α is the learning rate, and $L_{T}$ represents the task-specific loss function. MAML begins by training a general model across a variety of tasks to learn an initial set of parameters. These parameters are then fine-tuned for specific tasks through one or a few gradient descent steps, allowing the model to adapt quickly to new tasks from minimal data. MAML’s capacity for quick adaptation from minimal data proves invaluable in agricultural settings, where operational conditions and task requirements can rapidly change due to environmental factors (Finn et al., 2017). Despite its computational demands, MAML’s potential to facilitate swift adaptation with few examples aligns perfectly with the needs of the agricultural sector. This approach equips robotic systems with the flexibility required for precise monitoring and management in diverse agricultural scenarios, reinforcing the essential role of advanced learning techniques in evolving farming practices (Grant et al., 2018).

Offline imitation learning stands out as another strategic approach to bolstering the robustness of models, especially in contexts where engaging directly with experts is challenging. This method is instrumental in addressing the scarcity of data by leveraging a combination of precise expert policy state-action pairs alongside expansive datasets featuring less-than-ideal or suboptimal behaviors. Such a blend enriches the training material, presenting both ideal scenarios and common errors or variations (Zhang W. et al., 2023). The core of offline imitation learning lies in refining a dynamics model, which is taught to recognize and correct deviations from desired actions by imposing increased penalties on inaccurately represented segments of the state-action space (Chang et al., 2021). For agricultural applications, where diverse conditions and unpredictable variables make collecting comprehensive and optimal practice data difficult, offline imitation learning proves particularly useful for handling diverse and suboptimal datasets. For example, Liu et al. (2021) propose a curriculum offline imitation learning strategy that mitigates the drawbacks of mixed-quality datasets by using an adaptive experience-picking strategy to improve policy learning. Similarly, DeMoss et al. (2023) introduce DITTO, an algorithm that uses world models to address covariate shifts without additional online interactions, which is crucial for applications in unpredictable environments like agriculture. Moreover, Zhang W. et al. (2023) present a discriminator-guided model-based offline imitation learning framework that enhances performance by distinguishing between expert and suboptimal data, improving model robustness. Additionally, Park & Yang (2022) discuss the benefits of leveraging suboptimal datasets alongside optimal data, demonstrating that this approach can significantly improve learning outcomes even when expert data is scarce. By incorporating these insights, offline imitation learning proves to be a highly effective strategy for training agricultural robots, making them more adaptable and reliable in real-world scenarios.

Finally, the development of implicit BC models can also address the need for decisiveness in agricultural robots, which is essential for tasks requiring transitions between different types of crops or soil conditions. By framing BC as an energy-based modeling problem, where the model outputs a value indicative of action appropriateness, these models are capable of representing discontinuities effectively (Florence et al., 2022):

$$E_{BC}\left(x,a\right)=-\log P\left(a|x\right)$$ (6)

Here, in Equation 6, $E_{BC}$ signifies the energy associated with taking action a given observation x , with log⁡P(a|x) representing the probability of the action being expert-like. Studies have shown that implicit behavioral cloning policies, particularly energy-based models, often outperform explicit models in various robotic policy learning tasks, including those with high-dimensional action spaces and visual image inputs (Balesni et al., 2023). These models have demonstrated the ability to handle complex, discontinuous functions and improve policy learning in scenarios where traditional methods struggle (Singh et al., 2023).

In summary, incorporating a diverse array of strategies to mitigate covariate shifts and enhance model adaptability is crucial for advancing the field of agricultural robotics. Our comparative analysis, detailed in Table 1, showcases a spectrum of aforementioned methodologies, each offering unique benefits and challenges. This comparative insight is to guide future research and application development, ensuring the selection of strategies that not only address the inherent challenges of agricultural tasks but also leverage the full potential of behavioral cloning technologies.

**TABLE 1:** Comparative analysis of strategies to overcome covariate shift and improving BC model adaptability in agricultural application.

Strategy	Key characteristics	Advantages	Limitations	Agricultural applications	References
Interactive Imitation Learning (IIL)	Dynamically collects training data based on the model’s performance. Integrates expert feedback iteratively	Continuous model improvement. Reduces training-testing gap	High expert dependency. Cognitive load on experts	Precision irrigation. Autonomous pest control	Ross et al., 2011; Finn et al., 2017
Expert Policy Support Estimation	Estimates the expert policy’s support to guide learning. Uses reward shaping based on expert behavior	Enhances model fidelity. Tailorable to specific tasks	Requires deep expert behavior insight. Difficult to generalize	Crop monitoring. Yield optimization	Reddy et al., 2019
Constrained Operational Domains	Defines safe operational boundaries. Uses task-specific constraints	Increases safety and predictability. Reduces novel state risks	May restrict operational flexibility. Requires extensive domain knowledge	Ground vehicle navigation. Drone surveillance	Dadashi et al., 2020; Kim et al., 2023
Synthetic Data Augmentation	Uses artificial data to simulate out-of-distribution states. Prepares model for a wider range of scenarios	Enhances model robustness without real-world data. Tailorable to specific challenges	Risk of unrealistic data. Requires careful design	Adverse weather simulation. Rare pest outbreak training	Bansal et al., 2018; Wong et al., 2022
Implicit BC Models	Frames BC as an energy-based problem. Outputs a value indicating action appropriateness	Effective in representing discontinuities. Decisive in varied conditions	Complex model interpretation. Requires careful tuning	Crop type transitions. Soil condition adaptations	Florence et al. (2022)
Offline Imitation Learning	Utilizes both expert and suboptimal behavior data. Trains a dynamics model to apply penalties in poorly represented areas	Enhances model robustness in limited data scenarios. Mitigates overfitting to export data	Balancing expert and suboptimal data is challenging. Risk of learning from suboptimal actions	Data-scarce agricultural practices. Complex task learning	Chang et al. (2021)
Causal Misidentification	Identifies true causal relationships behind expert actions. Employs targeted interventions to refine accuracy	Improves model adaptability to dynamic conditions. Ensures training on the causal structure of tasks	Requires identification of accurate causal relationships. Potentially high complexity in modeling	Understanding crop growth factors. Pest control decision-making	De Stefano (2019)
Model-Agnostic Meta-Learning (MAML)	Optimizes model for quick adaptability to new tasks with minimal data	Rapid adaptation to varied conditions. Model-agnostic, broad applicability	Computationally intensive. Requires diverse training tasks	Rapid crop type adaptation. Environmental condition adjustments	Finn et al., 2017; Grant et al., 2018

#### 3.2.2 Inverse reinforcement learning

Inverse Reinforcement Learning (IRL) marks a significant advancement in computational learning sciences, emphasizing the extraction of underlying objectives or “rewards” that guide expert decisions, beyond just mimicking observed actions as BC. This focus is particularly crucial in agriculture, where decision-making incorporates complex variables that standard models may overlook. IRL excels by deducing the reward functions that experts implicitly follow, providing deeper insights into their decision drivers. At its core, IRL aims to uncover the ‘why’ behind the ‘what’ by analyzing expert actions to discover the hidden rewards that dictate such behaviors. Contrasting with Behavioral Cloning, which directly maps observations to actions, IRL delves into replicating the decision-making process itself. This distinction is critical in situations where direct observation alone does not clearly suggest the best action to take (Ng and Russell 2000).

The primary focus of IRL is on the reward function, R(s) , which assesses the desirability of a state s . In practical terms, especially within agricultural applications, the explicit reward function is hard to customize. As outlined in 7, the objective of IRL is to reverse-engineer this function from observed behaviors, effectively tackling the inverse of the usual reinforcement learning problem, where the goal is to learn the reward function that explains the observed behaviors (Arora and Doshi, 2021). Equation 7, highlited the mentioned approach.

Given: observed behaviors→Find: R(s) that explains these behaviors (7)

In IRL, the observed behaviors are utilized to infer the underlying reward structure. For instance, Malik et al. (2018) introduced cooperative inverse reinforcement learning (CIRL), which formalizes the value alignment problem as a game between a human and a robot. In CIRL, the robot learns the human’s reward function through interaction, leading to better alignment with human intentions. Also, Korsunsky et al. (2019) demonstrated the application of IRL in contextual Markov Decision Processes (CMDPs), where the reward function is inferred based on varying contexts. Their work highlighted the ability to generalize across different contexts by learning a mapping from contexts to rewards, which is particularly useful in dynamic agricultural settings where environmental conditions and tasks can vary significantly. Additionally, Lindner et al. (2022) proposed an active exploration strategy for IRL, which enhances the efficiency of learning the reward function by selectively querying the most informative regions of the state space. This method significantly improves the sample efficiency and the accuracy of the inferred reward functions, making it highly applicable to agricultural robotics where data collection can be expensive and time-consuming. By focusing on the underlying reward structures, IRL provides a more nuanced and flexible approach to understanding and replicating expert behaviors, thus enhancing its applicability in dynamic and complex environments (Shao and Er, 2012).

The scope of IRL’s application extends to multi-agent settings, demonstrating its scalability and the ability to infer reward functions that accurately generalize across varied settings and interactions. This broadens the potential of IRL in complex, interactive environments. Tailored IRL algorithms for continuous state spaces that provide formal guarantees on sample and time complexity are crucial for the development of reliable, automated control systems in precision agriculture (Fu et al., 2021; Dexter et al., 2021). Moreover, the challenge of model misspecification in IRL, especially significant in complex human-involved environments, is being addressed through rigorous mathematical analysis. This enhances the robustness of IRL models, ensuring the reliability of inferences drawn from observed behaviors. The introduction of innovative approaches that transcend the limitations of generative models, encoding versatile behaviors through iteratively trained discriminators, showcases the exceptional generalization capabilities of IRL. Such advancements underscore the diversity of expert behaviors and strategies that can be captured and implemented in autonomous agricultural systems (Skalse and Abate, 2023; Freymuth et al., 2021).

#### 3.2.3 Imitation from observation

IL typically requires both observing the states and the actions demonstrated by an expert. This dual requirement often necessitates comprehensive data collection, which can be restrictive and labor-intensive, especially in complex environments like robotics and gaming (Edwards et al., 2019). Experts might need to operate robots directly or use specialized software for recording actions in gaming, which not only demands significant effort but also limits the data to artificial conditions (Hu et al., 2022). Imitation from Observation (IfO) simplifies this by learning policies from state transitions alone, eliminating the need for action data (Liu et al., 2018). This approach mirrors natural learning processes seen in humans and animals, who can learn complex behaviors just by watching. IfO, therefore, broadens the scope of IL, making it possible to utilize diverse and previously inaccessible resources such as online instructional videos (Aytar et al., 2018).

IfO discards the necessity for direct action observation and instead utilizes state-only demonstrations. It leverages advanced machine learning algorithms, including deep learning and computer vision, to parse and interpret raw visual data (Choe et al., 2021). The foundational model for IfO can be formalized in Equation 8 by considering a set of state transitions observed from an expert’s demonstration:

$$s_{t+1}=f\left(s_{t},a_{t};\theta \right)$$ (8)

Where $s_{t}$ and $s_{t+1}$ are consecutive states, $a_{t}$ is the expert’s action which is typically unobserved in IfO, and θ represents the parameters of a model learned from data.

The key to IfO is the derivation of an optimal policy π that maximizes the expected reward over a trajectory, formalized under the reward function R derived from the state transitions as presented in Equation 9:

$$\pi^{*}=\arg\underset{\pi }{\max}E\left[\overset{T}{\underset{t=1}{\sum }}R\left(s_{t},\pi \left(s_{t}\right)\right)\right]$$ (9)

In the IfO framework, consecutive states $s_{t}$ and $s_{t+1}$ are modeled by the transition function f with parameters θ , where the action $a_{t}$ at time t , typically unobserved, influences the policy π that aims to maximize the cumulative reward over a trajectory from t=1 to T , calculated as the expected sum of rewards $R\left(s_{t},\pi \left(s_{t}\right)\right)$ , which assess the suitability of the actions recommended by π at each state. Liu et al. introduced an approach that utilizes context-aware translation models to adapt the expert’s demonstrations from an extrinsic viewpoint to the learner’s intrinsic perspective (Liu et al., 2018). This method essentially involves a function f that translates the context of input observations s from the demonstration: $s^{\prime }=f\left(s;\theta \right)$ . Here, $s^{\prime }$ is the state as perceived in the learner’s context, enabling the learner to interpret and mimic the expert’s behavior effectively.

In agriculture, IfO has the potential to revolutionize various practices by automating complex tasks such as planting, weeding, and harvesting. Robots equipped with IfO capabilities can learn from videos showing human farmers performing these tasks, thereby adapting human dexterity and judgment to robotic systems. For instance, in automated crop harvesting, IfO can facilitate the development of robotic systems that observe and learn the optimal techniques for harvesting specific crops. This application involves not only recognizing crop maturity via visual cues—akin to the capabilities developed through TCN (Time-Contrastive Networks) methods (Sermanet et al., 2018)—but also mimicking the motion patterns used by human harvesters to minimize damage to both the produce and the plant.

Despite its potential, IfO faces significant challenges, particularly in terms of data quality and environmental variability. The successful implementation of IfO depends heavily on the availability of high-quality, representative training data, which in many agricultural settings, must be captured across varying conditions to ensure robustness (Raychaudhuri et al., 2021). Additionally, the alignment of demonstrations across different contexts and the generalization of learned behaviors to new, unseen environments continue to pose considerable hurdles (Jaegle et al., 2021).

### 3.3 Advanced methods

#### 3.3.1 Adversarial imitation learning

Adversarial Imitation Learning (AIL) offers an efficient and advanced approach in the domain of artificial intelligence, particularly in the context of replicating expert behavior in complex environments. Traditional inverse Reinforcement Learning (IRL) often struggles with the computational complexities that arise when applied to large, complicated environments. These traditional methods are computation-heavy because they typically require solving a reinforcement learning (RL) problem as a subtask, which is both resource-intensive and slow. AIL simplifies this by eliminating the need to solve the RL problem in detail at every iteration, thereby reducing the computational demands significantly (Ho et al., 2016; Finn et al., 2016).

At the heart of AIL is an adversarial framework that orchestrates a dynamic game between two entities: an agent and a discriminator. The discriminator’s role is to differentiate between trajectories generated by the agent and those demonstrated by the expert, while the agent strives to produce trajectories indistinguishable from the expert’s, thereby incrementally refining its behavior towards an optimal policy that mirrors expert behavior. This adversarial process, inspired by the principles underlying Generative Adversarial Networks (GANs), has been instrumental in the methodological innovations and advancements in AIL (Deka et al., 2023). One of the landmark methodologies in AIL, Generative Adversarial Imitation Learning (GAIL), leverages a discriminator network to differentiate between the behaviors of the agent and the expert, using the confusion of the discriminator as a reward signal to guide the agent toward expert-like behavior. This approach, along with subsequent enhancements, has significantly improved sample efficiency, scalability, and robustness in AIL applications (Fu et al., 2017). Equation 10, highlighted the mentioned approach.

$$\text{Minimize: }L=\sum {\left(\text{action by agent}-\text{action by expert}\right)}^{2}$$ (10)

Where L is the loss indicating the difference in actions between the agent and the expert, minimized throughout training.

Recent advancements in AIL have focused on addressing practical challenges such as training instability and reward bias. The introduction of Support-weighted Adversarial Imitation Learning (SAIL) extends traditional AIL algorithms with expert policy support estimation, which enhances the quality of reinforcement signals and improves both performance and training stability across a variety of control tasks (Wang et al., 2020). This improvement potentially applies to agriculture by enhancing robotic precision in tasks such as planting, weeding, and harvesting, thereby increasing efficiency and reducing resource usage. Similarly, the development of Sample-efficient Adversarial Imitation Learning utilizes self-supervised representation learning to generate robust state and action representations, significantly improving the performance of imitation learning with reduced sample complexity (Jung et al., 2023). In agriculture, this could mean rapidly training models to recognize and act upon diverse crop and soil conditions, even with limited training data, thus enhancing adaptability and responsiveness to environmental changes. Orsini et al. (2021) emphasize the importance of various factors that contribute to the effectiveness of AIL, such as the balance between the generator and discriminator, and the stability of training processes. Their findings suggest that addressing these factors can significantly improve the performance and reliability of AIL methods. Furthermore, the integration of AIL with other learning paradigms, such as hybrid models that leverage the strengths of both AIL and traditional learning approaches, offers promising avenues for addressing broader social challenges and contributing to the global good. This multidisciplinary approach highlights the adaptability and versatility of AIL in various domains, from autonomous driving, where safety and ethical accountability are paramount, to healthcare, where AIL can enhance patient care simulation and treatment planning (Torabi et al., 2018; Yu et al., 2020).

#### 3.3.2 Diffusion policy

As mentioned before, IL in agricultural robotics aims to teach machines to emulate human actions or procedures demonstrated under various operational scenarios. The Diffusion Policy, a novel approach based on conditional denoising diffusion processes, innovatively extends visuomotor policy learning to complex, high-dimensional action spaces typical in agriculture, such as autonomous harvesting or precision farming (Chi et al., 2023). This technique notably surpasses traditional methods by its robust ability to handle multimodal action distributions and to maintain stability during training (Ho et al., 2020).

The Diffusion Policy is grounded in a generative model framework that iteratively refines predictions through a sequence of stochastic corrections, fundamentally based on Stochastic Langevin Dynamics. This process termed the conditional denoising diffusion process, involves an intricate computation of the gradient of the action-distribution score function. Such a calculation not only facilitates the handling of multimodal and high-dimensional action distributions but also enhances the learning efficiency and adaptability required for complex agricultural tasks (Song and Ermon, 2019; Chen, 2023). The denoising process can be mathematically represented in Equation 11 as follows:

$$x_{k-1}=x_{k}+\eta \nabla_{x}\log p_{k}\left(x_{k}|x_{0}\right)+\epsilon_{k},$$ (11)

Where $\epsilon_{k}∼N\left(0,\sigma^{2}I\right)$ is the Gaussian noise, η is the step size, and $\nabla_{x}\log p_{k}\left(x_{k}|x_{0}\right)$ is the gradient of the log-probability of the action given the initial state, computed at step k .

Traditional imitation learning approaches are often limited by the lack of flexibility in handling the diversity of agricultural environments and tasks. The Diffusion Policy addresses these limitations effectively:

• Expressing Multimodal Action Distributions: By leveraging the calculated gradient of the action score function and employing Stochastic Langevin Dynamics for sampling, the Diffusion Policy can proficiently represent and navigate through complex, multimodal action distributions. This feature is crucial for performing varied tasks where the environmental variability demands a high degree of adaptability in action planning (Chen, 2023; Neal, 2011).

• Adaptation to High-dimensional Action Spaces: The inherent capability of diffusion models to scale to high-dimensional spaces allows the policy to infer comprehensive sequences of actions. This scalability is essential for extended operations across large agricultural fields, where tasks may involve complex maneuvering and extended sequences of actions (Dosovitskiy et al., 2020; He et al., 2016).

• Stable Training Regime: Unlike traditional models that often face training instabilities due to the complexities of energy-based sampling methods, the Diffusion Policy stabilizes the training process by directly optimizing the gradient of the energy function. This methodological improvement is vital for maintaining consistent performance in the highly variable conditions typical of agricultural settings (Du et al., 2020; Gupta et al., 2019).

Implementing the Diffusion Policy within agricultural robotics involves several technical considerations to optimize its effectiveness in real-world settings:

• Precision Task Execution: The model’s high-dimensional action inference capability, combined with its robust training stability, allows for precise and efficient task execution. This precision is particularly beneficial for agriculture tasks that require exact movements, such as selective harvesting and precision pesticide application.

• Autonomous Navigation: Given its proficiency in handling complex distributions and high-dimensional data, the Diffusion Policy is ideally suited for autonomous navigation tasks in agriculture. This includes adapting to varying terrain types, crop densities, and other environmental factors that typically challenge traditional navigation systems.

• Integration with Robotic Systems: The practical application of the Diffusion Policy also involves its integration with existing robotic systems, which may require modifications to the robots’ sensory and processing units to fully leverage the capabilities of the policy (Urain et al., 2023; Karras et al., 2022).

The deployment of the Diffusion Policy in agricultural fields brings forth challenges that stem from the need for robust data collection, handling of environmental variability, and integration with diverse robotic technologies. Moreover, the adaptation of this policy to meet the specific demands of agricultural environments requires further empirical studies and technological innovations (Zeng et al., 2021; Zhang et al., 2020). The Diffusion Policy significantly advances imitation learning for agricultural applications, offering solutions that enhance the robustness, versatility, and precision of robotic tasks. This technology holds the potential to revolutionize agricultural practices by improving operational efficiency and reducing labor needs, paving the way for future innovations in robotic farming (Chi et al., 2023; Pearce et al., 2023).

Tables 2, 3 provide a summary of the critical analysis, emphasizing the technical capabilities and limitations of primary and advanced imitation learning models, respectively. These tables highlight how each model performs in the context of dynamic agricultural environments, as discussed in this section.

**TABLE 2:** Critical comparison of primary imitation learning models in agricultural applications, highlighting key aspects of scalability, adaptability, and performance.

Aspect	Behavioral cloning (BC)	Inverse reinforcement learning (IRL)	Imitation from observation (IfO)
Scalability	Efficient with structured, large datasets but necessitates diverse data to prevent overfitting (Ross et al., 2011)	Scales effectively in data-rich environments, demanding high computational resources (Arora and Doshi, 2021)	Demonstrates scalability with extensive visual or sensor data (Liu et al., 2018)
Adaptability	Quickly adapts to environments similar to its training data but struggles with novel scenarios (Kim et al., 2023)	Adapts to new tasks by learning underlying objectives, though re-derivation can be cumbersome (Arora and Doshi, 2021)	Shows adaptability in familiar settings but faces limitations with highly divergent new states (Hu et al., 2022)
Performance	Excels in environments resembling the training dataset but limited in dynamic conditions (Pomerleau, 1988)	Offers robust performance across varied scenarios, heavily dependent on precise reward estimation (Shao and Er, 2012)	Achieves high accuracy with well-modeled state transitions but is vulnerable if transitions are not accurately captured (Liu et al., 2018)
Generalization	Generalizes effectively within the scope of its training data but is constrained by limited diversity (Ross et al., 2011)	Excellent potential for generalization, reliant on the universality of inferred rewards (Skalse and Abate, 2023)	Generalizes well from observed to similar unobserved states, limited by the diversity of states during training (Edwards et al., 2019)
Dynamic Handling	Effective in dynamic settings if continuously updated with new data, but requires frequent retraining (Park and Yang, 2022)	Handles dynamic environments by learning applicable rewards but requires complex recalculations (Freymuth et al., 2021)	Capable in dynamic settings if regularly updated with new data, though demands continuous retraining (Liu et al., 2018)

**TABLE 3:** Critical comparison of advanced imitation learning models in agricultural applications, focusing on adaptability, performance, and dynamic handling.

Aspect	Adversarial imitation learning (AIL)	Diffusion policy
Scalability	Initial resource intensity decreases as more diverse data becomes available, enhancing scalability (Ho et al., 2016)	Handles high-dimensional action spaces effectively, though computational demands may constrain scalability (Chi et al., 2023)
Adaptability	Highly adaptable via its adversarial process, yet maintaining balance and training quality is crucial (Finn et al., 2016)	Adapts proficiently to complex action distributions but requires significant retraining for new task types (Chi et al., 2023)
Performance	Can outperform other models by learning nuanced behavior patterns but may experience instability during early training phases (Ho et al., 2016)	Excels in handling multimodal actions; however, its performance consistency heavily relies on the quality of stochastic modeling (Ho et al., 2020)
Generalization	Provides strong generalization across diverse scenarios when adequately trained, though there is a risk of overfitting (Fu et al., 2017)	Proficient at generalizing across high-dimensional and multimodal environments, necessitating thorough training (Song and Ermon, 2019)
Dynamic Handling	Adapts dynamically through continuous adversarial training, necessitating consistent data input and adjustments (Finn et al., 2016)	Manages dynamic changes efficiently through stochastic corrections but requires ongoing calibration (Song and Ermon, 2019)

## 4 Current applications of IL in agricultural and biological engineering

In the preceding section, we discussed various imitation learning methods and techniques, detailing their potential to significantly enhance agricultural practices. Despite the advantages that imitation learning offers to the field, its application within agriculture remains relatively nascent, with few research endeavors exploring its full potential. This section delves into the most recent research in this area, explores how these innovations can be expanded and suggests new subjects for future investigation.

**FIGURE 4:**
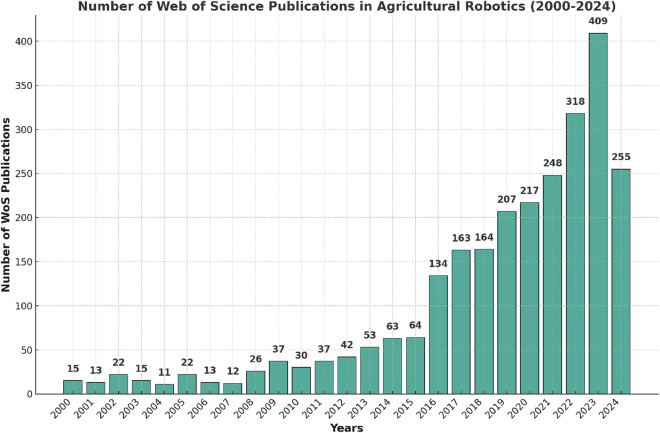
Annual number of publications in agricultural robotics indexed by web of science (2000-2024).

Recent studies reveal a rising interest in bringing advanced technologies like robotics and AI into agriculture, especially when it comes to using IL to boost efficiency and precision in farming. In the comprehensive review by Starostin et al. (2023), the authors highlight the increasing global focus on developing robotic platforms for agricultural purposes, particularly in countries like the United States, China, and Germany, where research and development in this field have accelerated in recent years. The updated findings from this research are illustrated in Figure 4.

**FIGURE 5:**
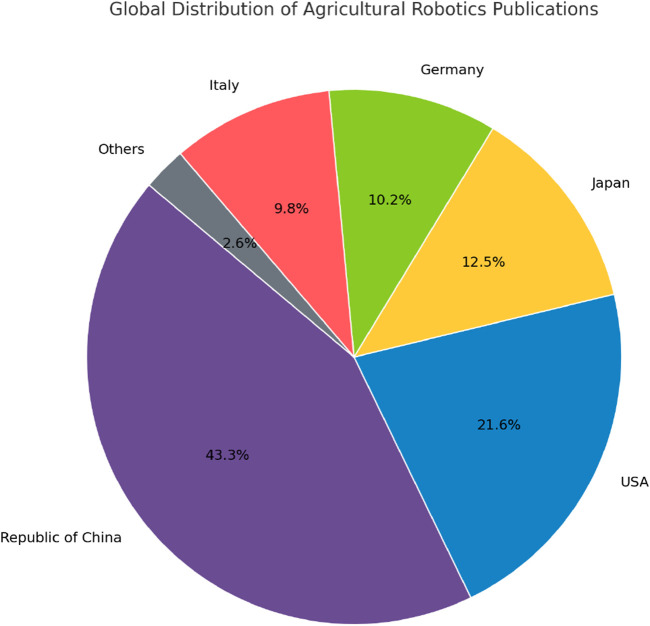
Global distribution of agricultural robotics publications.

The Figure 5 provided illustrates the global distribution of research publications in agricultural robotics, highlighting the significant interest and investment in this field by various countries. Notably, the Republic of China leads with 43.3% of the total publications, indicating a strong commitment to advancing robotic farming technologies. The United States follows with 21.6%, while Japan, Germany, and Italy also show considerable contributions. These countries are not only at the forefront of research but have also established or are developing policies to support the implementation of these innovations. To ensure the strategic development of agricultural robotics, future policies in key countries are expected to focus on enhancing research in intelligent robotics, fostering innovation in sensor and automation technologies, and supporting the integration of robotics into large-scale agricultural production systems (Zhao et al., 2020).

As we look to the future, with policies aiming to speed up the adoption of advanced technologies in agricultural robotics, it becomes more important than ever to explore new and innovative ways to train these systems effectively. The early application of imitation learning in agriculture, as demonstrated by Dyrstad et al., where robots were instructed to grasp real fish, illustrates the substantial potential of utilizing virtual reality as a sophisticated training tool (Dyrstad et al., 2018). This innovative approach could be effectively expanded to encompass a range of complex agricultural tasks, particularly with the daily improvement of Machine Learning methods in agricultural product prediction (Khan et al., 2020). These improvements, combined with predictive models for agricultural production, offer valuable tools for policymakers and farmers to enhance fruit production, optimize resource management, and address the growing demand for agricultural efficiency (Rehman et al., 2018). For instance, it could be adapted for the harvesting of irregularly shaped or delicate fruits and vegetables, thereby opening new avenues for research in adaptive robotic handling techniques. Additionally, the implementation of imitation learning holds considerable promise for challenging environments such as poultry plants or crab meat harvesting operations. These fields, characterized by their harsh working conditions, could greatly benefit from the precision and adaptability that imitation learning-equipped robots can offer, potentially transforming labor-intensive processes into more efficient, automated operations.

Kim, Ohmura, and Kuniyoshi (2022a) made significant strides in the robotic handling of delicate tasks with the development of a sophisticated robotic system for peeling bananas. This system employs goal-conditioned dual-action deep imitation learning and features a dual-arm robot setup, where one arm stabilizes the banana while the other expertly peels it. This complex coordination between the arms is crucial for handling tasks that require precision and gentle handling, demonstrating the potential for similar robotic systems to be adapted for processing other agricultural products that necessitate delicate operations, such as peeling or slicing, thus minimizing waste and maximizing efficiency.

Complementing this, Kim J. et al. (2022) introduced the Deep-ToMaToS, a deep learning network tailored for the 6D pose estimation of tomatoes, enabling precise robotic harvesting based on maturity classification. This approach highlights the capability of deep learning models to significantly enhance the accuracy and efficiency of robotic systems in agriculture, especially in recognizing and classifying crops according to their maturity. Together, these studies showcase the potential of advanced machine learning models to revolutionize the automation of intricate agricultural tasks, from the precise handling and processing of individual fruits to the efficient harvesting of crops, opening avenues for these technologies to be applied across a broader spectrum of agricultural needs.

The research conducted by Tsai et al. (2018) in both 2018 and 2019 significantly advanced the precision and adaptability of agricultural robots through the development of visual picking controls using end-to-end imitation learning. In their 2018 study, Tsai et al. (2019) focused on enabling a 6-DoF (Degree of Freedom) manipulator to adapt its picking strategies based on real-time visual cues. This approach allowed the robot to effectively identify and pick objects in a dynamic environment, which is crucial for applications such as fruit or vegetable harvesting where conditions can vary widely. Extending these capabilities, another research introduced an omnidirectional mobile manipulator equipped with enhanced multi-task learning capabilities. This system was not only capable of navigating across different terrains but could also perform various agricultural tasks simultaneously, thereby increasing efficiency and reducing the need for human intervention.

These enhancements in robotic technology suggest that the methods developed by Tsai et al. could be applied to a broader range of agricultural robots, particularly those used for seeding and weeding. By adapting the visual picking controls and multi-task learning approaches, these robots could potentially operate autonomously within fully automated farm environments. Such advancements could lead to the creation of multi-robot systems where various agricultural tasks, from planting to pest control, are allocated efficiently among different robots. Each robot could learn from human experts through imitation, thereby continually improving its operational efficiency and adaptability.

Moreover, the foundational work by Tsai et al. opens up opportunities for future research into the collaborative operation of these robots in a coordinated system. This could involve the development of algorithms that enable robots to learn not only from human demonstrations but also from each other, optimizing task allocation and execution in real-time based on environmental conditions and ongoing learning. Such systems would represent a significant step toward the realization of smart agriculture ecosystems, where multiple robotic agents work in concert to manage and optimize various farming operations, leading to higher productivity and sustainability.

Misimi et al. (2018) and Porichis et al. (2023) both explore the capabilities of robotic systems in handling specific agricultural products, emphasizing the importance of delicate handling to maintain product integrity and quality. Misimi et al. (2018) developed robust learning-from-demonstration techniques, initially focused on the handling of fish and other malleable materials. This approach utilizes visual and tactile feedback to adapt to the varied textures and consistencies encountered in these materials, which is crucial for tasks such as filleting or packaging in food production environments. The techniques demonstrated by Misimi et al. (2018) have significant potential for extension to the handling and processing of plants, particularly in applications where the preservation of structural integrity is critical, such as in the handling of soft fruits and vegetables. On the other hand, Porichis et al., 2023 addressed the specific challenges associated with mushroom harvesting by developing a visual imitation learning system that not only recognizes mushrooms but also picks them with precision. Their system is designed to minimize damage to the mushrooms, thereby improving yield and product quality. The advanced visual recognition capabilities and delicate handling methods are particularly important for crops like mushrooms, which are susceptible to bruising and damage during the picking process. Both studies underscore the broader applicability of these robotic technologies to other types of crops that require gentle handling. By adapting the visual and tactile-based imitation learning techniques developed in these studies, it is conceivable to design robotic systems that could handle a wider array of delicate agricultural products, from berries to leafy greens, reducing labor requirements and enhancing product quality. These advancements hold the promise of transforming agricultural practices by introducing more efficient, precise, and gentle robotic systems into the harvesting and processing stages, ultimately leading to more sustainable and productive agricultural operations.

Zhou et al., 2022a reviewed the latest advancements and persistent challenges in the development of robotic systems for fruit harvesting. This paper underscores the critical need for robots that are not only intelligent but also highly adaptive, capable of handling the diverse and often unpredictable conditions typical of agricultural environments. The authors emphasize the complexity of accurately identifying and harvesting different types of crops, which vary widely in size, shape, and the degree of delicacy required during handling. The study encourages further research into integrating multi-sensor fusion techniques and artificial intelligence to improve the autonomy and efficiency of harvesting robots. By doing so, these machines could potentially learn from each harvest, optimizing their paths and techniques based on accumulated experiences and data, thereby reducing waste and increasing yield. This emphasis on the need for greater intelligence and adaptability in robotic harvesting systems calls for a multidisciplinary approach, combining robotics, machine learning, and agricultural sciences to develop solutions that can sustainably meet the demands of global food production.

Given the nascent state of imitation learning in agriculture, as evidenced by the literature, there are ample opportunities for future investigations. These studies could focus on developing more sophisticated models of learning that not only imitate simple actions but also complex decision-making processes, thereby enhancing the cognitive capabilities of agricultural robots. As agricultural demands evolve alongside technological advancements, imitation learning is poised to become a pivotal technology in transforming agricultural practices, addressing some of the most pressing challenges in the field.

## 5 Challenges and ethical considerations

As agricultural robotics and IL evolve, they present a multitude of challenges and ethical considerations that necessitate thoughtful deliberation and proactive management. This chapter aims to highlight the primary obstacles in the deployment of IL technologies in agriculture, address the ethical implications, and propose directions for future research and development.

### 5.1 Data acquisition and quality

A major hurdle in implementing IL in agricultural robotics is securing high-quality data (Yang et al., 2021). The process of gathering substantial volumes of annotated data in agricultural settings is notably laborious and time-consuming, demanding considerable human expertise. Moreover, the quality of data can be compromised by human errors due to distractions or limited visibility and comprehension of complex environments (Wu et al., 2019; Sasaki and Yamashina, 2020).

Crowd-sourced datasets, often employed to boost the robustness and efficacy of IL policies, introduce additional challenges due to their inherent variability. These datasets display a wide range of demonstration qualities, mirroring the varying expertise of contributors (Ramrakhya et al., 2022). While discarding lower-quality demonstrations might appear to be a straightforward fix, it is rarely feasible due to the massive manual effort required for data curation, which is typically impractical (Sasaki and Yamashina, 2020).

Furthermore, the diverse and often unstructured nature of agricultural environments—from open fields to greenhouses—poses unique challenges for data collection. The need for adaptable data collection strategies is accentuated by environmental variations such as lighting, weather, and seasonal changes, which can significantly affect the physical characteristics of crops and disrupt data consistency across different periods (Ramrakhya et al., 2022; Bechar and Vigneault, 2016). Adverse weather conditions like rain and fog can also impair the quality and reliability of sensor data. Moreover, the performance of commonly used sensors in agricultural robotics, including cameras, LiDAR, and GPS, may be hindered by limitations in resolution, range, and accuracy under challenging conditions such as dust, rain, and fluctuating light levels (Ozdogan et al., 2010; Bargoti and Underwood, 2017). These issues are critical as they directly influence the quality of data essential for training dependable IL models.

### 5.2 Generalization and adaptability

Ensuring IL models generalize effectively across diverse agricultural settings presents a significant challenge. Models trained in specific environments may not perform effectively when deployed in new or different settings due to variations in crop types, growth stages, and farming practices. This issue, often referred to as the “domain shift” problem, necessitates the development of models that can adapt in real time to dynamic and unpredictable agricultural environments.

Addressing domain discrepancies is a focal point of recent IL research, with studies exploring dynamics, viewpoint, and embodiment mismatches Kim et al. (2020); Stadie et al. (2017). These efforts typically involve developing mappings between state-action spaces to foster domain-invariant features, which are crucial for successful policy learning across varying conditions. The need for feature-level domain adaptation is discussed by Kouw et al. (2016), who propose an approach that models the dependence between two domains through a feature-level transfer model, aiming to reduce the feature distribution discrepancies that are common in diverse agricultural settings. Moreover, Nguyen et al. (2021) introduced TIDOT, a model that employs the principles of imitation learning and optimal transport for unsupervised domain adaptation. This model demonstrates how a “teacher” agent in the source domain can guide a “student” agent in the target domain, thereby enabling real-time adaptation to new agricultural environments. These advanced methodologies, including Cross-embodiment IRL (XIRL) and other approaches by Fickinger et al. (2021), focus on extracting task-specific policies that are resilient to variations in environment dynamics and embodiment (Beliaev et al., 2022; Kim et al., 2020).

### 5.3 Computational constraints

Addressing the computational constraints in deploying IL models on agricultural robots is crucial due to the limited computational resources and energy availability inherent to field robots. Balancing the performance of high-complexity models with the constrained computational power and battery life of agricultural robots is essential for practical applications.

To tackle these challenges, researchers have focused on model compression and optimization techniques that maintain model performance while reducing computational demands and energy consumption. Techniques such as quantization, pruning, and knowledge distillation are particularly effective in adapting IL models to the constraints of agricultural robots (Tokekar et al., 2016). These methods aim to decrease the model size and computational requirements, thus making IL models more suitable for deployment on resource-constrained robots in the field.

Liu et al. (2021) discussed the application of pruning and quantization to reduce the computational load of models without significantly sacrificing performance, making them feasible for implementation on robots with limited processing capabilities and battery life. Similarly, Tokekar et al. (2016) explored the balance between model complexity and computational feasibility, emphasizing the importance of efficient energy management in prolonging the operational time of field robots. Further research by Buchli et al. (2017) and McCool et al. (2016) has highlighted the critical role of energy-efficient model design in ensuring that robots can perform extended tasks without frequent recharging. These studies suggest that advanced model optimization strategies, beyond traditional compression techniques, are necessary to achieve the dual goals of high performance and low energy consumption in agricultural settings.

Recent developments in this area have also included more sophisticated approaches such as differentiable constrained IL for robot motion planning and control, which integrate hard constraints into the learning process to ensure safe and efficient robot operation under limited computational resources (Diehl et al., 2022).

### 5.4 Ethical and social considerations

The deployment of agricultural robots brings forth profound ethical and social considerations, including issues of labor displacement, data privacy, and the broader implications on societal structures. The integration of these technologies can significantly affect employment, amplify existing social inequalities through algorithmic biases, and challenge the governance frameworks needed to ensure safe and equitable use.

The discussion around the moral status of robots, as introduced by Bostrom and Yudkowsky (2018), emphasizes the potential for robots to alter societal norms and structures dramatically. They suggest that without careful consideration, the deployment of autonomous systems could exacerbate social inequalities and undermine human dignity by making biased decisions or behaving unpredictably in human environments. This necessitates robust ethical frameworks to ensure that robotics technologies are aligned with human values and safety standards.

As the agricultural sector transitions from labor-intensive methods to automation, the role of robots has expanded. This shift, discussed by Marinoudi et al. (2019), raises significant questions about the effects on employment and the social fabric of rural communities. As repetitive tasks such as fruit picking and weeding are increasingly automated, many workers may find their roles replaced by machines. Reskilling programs are urgently needed to help these workers transition to new roles, such as robot maintenance and operation. De Stefano (2019) suggests that without these interventions, rural communities may experience increased income disparities and social disruption. The introduction of automation can also widen the gap between large, technology-driven farms and smaller farms that lack the resources to adopt these innovations, potentially leading to socioeconomic inequality (Dauth et al., 2021).

In many cases also, agricultural roles may be augmented rather than entirely replaced, with robots starting to work alongside humans. This collaborative approach could mitigate some negative impacts on employment but requires careful management and supportive policies to ensure equitable outcomes. New job opportunties will also be created in technology-driven roles such as robot maintenance, programming, and system management. Dauth et al. (2021) observed that while robots have displaced certain jobs in manufacturing, they have also generated new, often higher-quality positions in the service sector. This suggests that educational and training programs are crucial in preparing displaced workers for new opportunities in an increasingly automated agricultural landscape.

The digitization of agriculture also brings to the forefront issues surrounding the privacy and security of farm data. As farming practices and crop management become more data-driven, the risk of sensitive information falling into unauthorized hands increases. Strong data security, anonymization protocols, and clear data governance structures are essential to protect farmers’ privacy and ensure that the collected information is used responsibly. Data ownership is a critical issue, especially as farmers increasingly rely on third-party platforms to analyze their data. When farmers outsource data processing to these platforms, they risk losing control over their data, potentially exposing them to exploitation or misuse. Rose et al. (2021) emphasize the importance of embedding ethical considerations early in the design and development phase of autonomous agricultural robots to safeguard data integrity and ensure farmer privacy. Strong data security measures, such as encryption and anonymization, are essential to protect farmers’ information. However, data ownership is a growing issue, especially as farmers rely on third-party platforms to analyze and process their data. When this information is uploaded to external systems, farmers risk losing control over it. Kshetri (2014) points out that data ownership policies need to be clearly defined, with specific regulations governing who has access and rights to the data collected by agricultural robots. Clear legal frameworks should ensure that farmers retain control over their data and that it cannot be misused by corporations or third-party platforms (Wolfert et al., 2017).

The societal impact of robotics in agriculture also extends beyond employment. Klerkx and Rose (2020) argue that automation may lead to land consolidation, as smaller farms struggle to compete and are forced to sell their land to larger agribusinesses. This shift could reduce the autonomy of smallholder farmers and weaken the social fabric of rural communities. It is essential that policymakers ensure equitable access to robotics and AI technologies to prevent this divide.

Addressing the ethical and social implications of agricultural robots requires effective policymaking that aims to distribute the benefits of automation broadly across society, supporting both displaced workers and the communities impacted by these technological changes. Sparrow and Howard (2020) emphasize the need for key policy choices that maximize the social, environmental, and economic benefits of agricultural robotics while mitigating potential harms. The integration of robotics in agriculture thus presents a complex landscape of ethical and social challenges, including potential labor displacement, the creation of new technological roles, concerns over data privacy, and the need for robust governance frameworks. A comprehensive and balanced approach is required to ensure that the advancement of robotics in agriculture aligns with human values and promotes equitable, socially just outcomes. This approach will be critical in navigating the evolving moral landscape as robotics become more embedded in daily farming operations.

## 6 Conclusion and future directions

This survey on imitation learning in agricultural robotics encapsulates the transformative potential and innovative impact of this technology in revolutionizing agricultural practices. IL adeptly mimics expert human strategies in complex and variable environments, enhancing efficiency, adaptability, and productivity in agricultural operations. Techniques such as AIL and Diffusion Policy in IL are significant advancements, in improving the practical deployment of robotic systems in farming.

Despite these advancements, the application of IL in agriculture faces substantial challenges including data collection in diverse environments, the need for model generalization across different agricultural contexts, and computational constraints limiting field deployment. Moreover, the ethical implications surrounding labor displacement and data privacy necessitate a balanced approach to technology integration.

Future research should address these challenges by enhancing model adaptability, improving data collection quality through advanced sensing technologies, and optimizing IL models for computational efficiency using techniques such as model compression and quantization. Additionally, developing comprehensive ethical frameworks and policy guidelines is crucial to address socio-economic impacts and ensure equitable benefits from agricultural robotics.

Exploring collaborative human-robot interactions and expanding IL applications to include a broader range of agricultural tasks, such as pest management and environmental monitoring, will also be pivotal. These efforts will not only mitigate job displacement concerns but will also enhance the acceptance and effectiveness of robotic technologies in agriculture.

By focusing on these areas, the future of IL in agricultural robotics can achieve its potential, leading to revolutionary advancements in agricultural practices. This will contribute to more sustainable, productive, and less labor-intensive farming methods globally, aligning technological progress with human values and environmental needs.
